# A New Visual Stimulation Program for Improving Visual Acuity in Children with Visual Impairment: A Pilot Study

**DOI:** 10.3389/fnhum.2016.00157

**Published:** 2016-04-18

**Authors:** Li-Ting Tsai, Jung-Lung Hsu, Chien-Te Wu, Chia-Ching Chen, Yu-Chin Su

**Affiliations:** ^1^School of Occupational Therapy, College of Medicine, National Taiwan UniversityTaipei, Taiwan; ^2^Occupational Therapy-Low Vision Rehabilitation, University of Alabama, BirminghamAL, USA; ^3^Section of Dementia and Cognitive Impairment, Department of Neurology, Chang Gung Memorial HospitalLinkou, Taiwan; ^4^Graduate Institute of Humanities in Medicine, Taipei Medical UniversityTaipei, Taiwan; ^5^School of Medicine, National Yang-Ming UniversityTaipei, Taiwan; ^6^Stroke Center and Department of Neurology, Taipei Tzu Chi Hospital, Buddhist Tzu Chi Medical FoundationNew Taipei City, Taiwan

**Keywords:** visual impairment, visual acuity, visual rehabilitation, visual stimulation, visual perceptual learning, attentional modulation, visual evoked potential

## Abstract

The purpose of this study was to investigate the effectiveness of visual rehabilitation of a computer-based visual stimulation (VS) program combining checkerboard pattern reversal (passive stimulation) with oddball stimuli (attentional modulation) for improving the visual acuity (VA) of visually impaired (VI) children and children with amblyopia and additional developmental problems. Six children (three females, three males; mean age = 3.9 ± 2.3 years) with impaired VA caused by deficits along the anterior and/or posterior visual pathways were recruited. Participants received eight rounds of VS training (two rounds per week) of at least eight sessions per round. Each session consisted of stimulation with 200 or 300 pattern reversals. Assessments of VA (assessed with the Lea symbol VA test or Teller VA cards), visual evoked potential (VEP), and functional vision (assessed with the Chinese-version Functional Vision Questionnaire, FVQ) were carried out before and after the VS program. Significant gains in VA were found after the VS training [VA = 1.05 logMAR ± 0.80 to 0.61 logMAR ± 0.53, *Z* = –2.20, asymptotic significance (2-tailed) = 0.028]. No significant changes were observed in the FVQ assessment [92.8 ± 12.6 to 100.8 ±*SD* = 15.4, *Z* = –1.46, asymptotic significance (2-tailed) = 0.144]. VEP measurement showed improvement in P100 latency and amplitude or integration of the waveform in two participants. Our results indicate that a computer-based VS program with passive checkerboard stimulation, oddball stimulus design, and interesting auditory feedback could be considered as a potential intervention option to improve the VA of a wide age range of VI children and children with impaired VA combined with other neurological disorders.

## Introduction

Impaired visual acuity (VA) can contribute to developmental problems and learning difficulties in perception, cognition, motor control, communication, and visual attention in children with visual impairment or children with developmental disabilities (Ferrell et al., 1998; Maurer et al., 2007). Various studies have provided evidence that visual training can improve these children’s VA (Sonksen et al., 1991; Leguire et al., 1992; Malkowicz et al., 2006; Polat et al., 2009; Alimovic and Mejaski-Bosnjak, 2011; Huurneman et al., 2013; Tsai et al., 2013). Among these training programs, visual stimulation (VS; Malkowicz et al., 2006; Alimovic and Mejaski-Bosnjak, 2011) and visual perceptual learning (VPL; Polat et al., 2009; Huurneman et al., 2013; Tsai et al., 2013) are two of the most important intervention strategies.

The VS approach has been widely used in education or clinical practice for several years to stimulate visually impaired (VI) infants or VI children who have additional disabilities or cognitive challenges and barely react to the visual environment (e.g., severe cerebral visual impairment, CVI; Bell, 1986; Powell, 1996). The main principles in applying the conventional VS addressed by Bell (1986) are to systematically and sequentially present visual stimuli according to a VI infant’s or a child’s visual function, such as by using lights, bright colors, or a higher order of high-contrast black-and-white patterns to motivate the VI infants or children to look and thereby to strengthen their neural pathways and facilitate their visual development (Powell, 1996). The types and properties of the VS parameters, such as light and its brightness, color, and contrast, or a pattern and its complexity, and the ways these parameters are used in the VS program are mainly based on the findings of visual development studies in infants (Powell, 1996).

Even though the VI children who were included into the VS studies had the same diagnosis (e.g., CVI), their visual capacities were usually heterogeneous. Therefore, various visual parameters, such as a flashlight in a darkened room, different spatial frequencies of square wave gratings, checkerboards of different sizes, or other geometric patterns are typically and simultaneously used in a single conventional VS study to stimulate children with various degrees of visual function (Leguire et al., 1992; Malkowicz et al., 2006; Alimovic and Mejaski-Bosnjak, 2011). Conventional VS programs also create a rich visual environment that agrees with the children’s visual abilities to encourage and increase their active visual behaviors, such as detecting, fixating, or orienting (Malkowicz et al., 2006). In addition to conventional VS programs, the VS approach is also used for children who suffer from visual field deficits due to cerebral lesions, but these children should be able to cooperate with systematic training involving visual field stimulation (Werth and Moehrenschlager, 1999).

In contrast, VPL requires a child with an ability to actively discriminate visual features through extensive exposure and training to optimize visual gains (Polat et al., 2009; Huurneman et al., 2013; Tsai et al., 2013). Both VS and VPL programs involve repeated and systematic training while emphasizing the operating features of visual parameters and attentional modulation to enhance the early stage of visual information processing (Grill-Spector and Malach, 2004; Poggel et al., 2004; Cohen-Maitre and Haerich, 2005). Despite a substantial body of evidence supporting the benefits of VPL (Sagi, 2011), it is less suitable for young children or children with additional disabilities such as mental or attentional deficits because it requires repetitive and monotonous discrimination and judgment of small perceptual differences between visual features for an extended period of time (Tsai et al., 2013). For these special children, the conventional VS method seems to be a more feasible approach than VPL.

Although the concept of conventional VS has been broadly used in clinical practice for VI infants or VI children with additional impairments (Bell, 1986), empirical evidence to validate its intervention effects as compared to VPL is still very limited (Leguire et al., 1992; Powell, 1996; Malkowicz et al., 2006; Alimovic and Mejaski-Bosnjak, 2011). Unlike a VPL program, the conventional VS program entails difficulties in quantifying VS doses, namely, the amount and intensity of VS (Malkowicz et al., 2006; Alimovic and Mejaski-Bosnjak, 2011), and few VS studies have investigated the dose of VS required to effect visual changes (Powell, 1996). In addition, the length of the intervention time of the VS programs is often long, lasting from several months to 1 year (Malkowicz et al., 2006; Alimovic and Mejaski-Bosnjak, 2011). As a result, it is hard to exclude the contributions of developmental factors or draw conclusions about the effects of VS on visual improvement. Therefore, research to improve the design of a conventional VS program, quantify the amount and intensity of VS, and examine the dose-response effects of VS are needed (Powell, 1996). Moreover, based on the research findings of visual science, early-stage visual processing—from basic contrast/color detection and contour/motion identification to early visual information integration—is affected by top-down modulation (e.g., attention; Gilbert and Li, 2013). Nevertheless, the effect of attentional manipulation on VS programs has been discussed infrequently in previous VS studies.

The purpose of this study was to design a new VS program by quantifying the dose of VS and manipulating the effect of top-down modulation into training. To address this issue, we designed a new computer-based VS training program and evaluated its effectiveness in improving the VA of children with impaired VA caused by deficits along the anterior (from the eyeball to the lateral geniculate body) and/or posterior (from the optic radiation to the visual cortex) visual pathways.

The visual stimuli used in the present study incorporated pattern stimuli (constant checkerboard pattern reversal as the core stimulus for passive stimulation) and oddball stimuli (random, infrequent, and salient stimuli for enhancing and maintaining attention). The checkerboard pattern is used extensively in visual science, visual function assessment, and visual training because it is relatively primitive, simple, and reliable (De Valois et al., 1979; Leguire et al., 1992; Bach and Ullrich, 1997). The rationale for adopting the oddball stimulus is based on the concept of the oddball paradigm, wherein observers respond to infrequent and irregular target stimuli within a series of standard stimuli (e.g., the checkerboard pattern reversal used in this study). The oddball paradigm is usually adopted within event-related potential research to elicit the P300 component, which is associated with cognitive processes (e.g., memory, attention, and executive function; Polich and Criado, 2006). Thus, a specific goal of the present study was to examine whether integrating passive stimulation (pattern reversal) and attentional modulation (oddball stimulus) in a computer-based VS program (quantifying the dose of stimulation) can improve the VA of children who have impaired VA with or without additional developmental problems. We simultaneously recorded the visual evoked potential (VEP) as a neurophysiological signature to examine whether the treatment effect of VS training was manifested by changes in the scalp potential evoked in the visual cortex. We hypothesized that the training effect of the current VS program might change the amplitude and/or latencies of the corresponding VEP (Kelly et al., 2014). In addition, functional vision was also evaluated to examine whether the VS training effect could be transferred to the performance of functional activity.

## Materials and Methods

All procedures were reviewed and approved by the Institutional Review Board of Taipei Tzu Chi Hospital, Buddhist Tzu Chi Medical Foundation (03-X04-009), and all tests were conducted in accordance with the tenets of the Declaration of Helsinki. Parents were informed of the study purpose and all measurement and training procedures before providing written consent for participation.

### Participants

Children with impaired VA were mainly referred from two private institutes: the Taipei Parents’ Association for the VI (an organization for people who are blind or VI) and the First Social Welfare Foundation (an organization for people who have sensory, physical, or intellectual disabilities). The inclusion criteria were: (1) visual impairment, not limiting the etiology of the impaired vision, and (2) VA of at least hand motion (HM), meaning that the participant could see hand movement at one foot. The exclusion criteria were (1) the inability to follow simple instructions, such as “look at me” or “pick up a toy for me,” (2) the inability to comprehend cause-and-effect relationships, and (3) the inability to maintain visual attention to a task for over 1 min, which was assessed by using toys that were interesting to the participants. The numbers and properties of the included participants are provided in **Table 1** and in the section “Participant Summary.”

**Table 1:** Participants’ demographic information (*n* = 6).

Subject	Sex	Age, y	Diagnosis		Correction
A1	M	1.2	ROP (stage 5 in OD and 4 in OS) and Aphakia (OU) and hearing impairment	OD	S: +8.75 C: –1.50 ax: 65
				OS	S: +14.50 C: –1.75 ax: 15
A2	F	2.3	Subdural hemorrhage with retinal hemorrhage in OD and retinal detachment in OS		No correction
A3	F	3.2	Suspected autistic disorder with amblyopia (OU)	OD	S: +3.50 C: –1.50 ax: 170
				OS	S: +3.25 C: –2.25 ax: 180
A4	M	3.9	Retinoschisis (OU)	OD	S: +8.00 C: –3.00 ax: 11
				OS	S: +6.00 C: –1.75 ax: 0
A5	F	5.3	Premature birth with amblyopia (OU)	OD	S: +0.75 C: –2.25 ax: 125
				OS	S:+0.75 C:–2.75 ax: 20
A6	M	7.8	Cerebral palsy with cerebral visual impairment	OD	S: +0.50 C: –2.00 ax: 180
				OS	S: +0.25 C: –2.75 ax: 50

*CP, cerebral palsy; CVI, cerebral visual impairment; RH, retinal hemorrhage; ROP, retinopathy of prematurity. OU, both eyes; OD, right eye; OS, left eye.*

### Apparatus

For the training session, the stimuli of black and white checkerboard patterns were generated by C# language running on an ASUS PC with an Intel Core i7 display card. The stimuli were displayed on a 19-inch ViewSonic G90fB CRT monitor with a resolution of 1,280 × 1,024 and a 75 Hz frame rate. Gamma was measured with a Minolta LS-110 luminance meter and corrected during the experiment (mean luminance = 27 cd/m^2^). In addition, an adaptive single switch was used to enable the children to actively participate in the training processing.

### Stimuli

The stimuli used in this study integrated constant checkerboard pattern reversal and random oddball stimuli. For the checkerboard pattern, the check size, reversal rate, and check contrast were designed according to each child’s VA, age, and performance during training. Manipulation of the check size (spatial frequency), contrast, and reversal rate in a checkerboard pattern is useful in a VS program. The initial check size (visual angle) was extracted through a transformation function based on each participant’s VA [decimal VA or spatial frequency (cycles per degree/cpd; Iyer et al., 2013)]:

$$\theta =\frac{1}{cpd}\times \frac{1}{\sqrt{2}},$$

where, visual angle𝜃 = tan^-1^(H/D); H, height of a single check; and D, the viewing distance. The VA was transformed into the units of cpd for initial check size estimation (Iyer et al., 2013).

For children with very severe VA of hand movement and finger counting, decimal VAs of 0.0052 and 0.010 were assigned (Lange et al., 2009). Because most of the children in the current study had severe visual impairment or were recruited from institutions for children with developmental delays, they usually did not use their residual vision well, so they needed a larger check size to attract their visual attention and to increase their fixation duration. Although the framework of VPL training suggests that the visual learning effect will be more efficient with stimuli that are close to the estimated size threshold (Huang et al., 2008), the actual training check sizes for the participants in this study were larger than the initial estimates. As the children became familiar with the training procedures and their responses stabilized, the check sizes were gradually reduced. We used checkerboard patterns with a reversal rate of 2 Hz for children younger than 3 years old or VA below 0.1, and of 4 or 5 Hz for other children. The contrast of the checkerboard pattern was set at 90 or 80% in the beginning. For children with better acuity (decimal VA > 0.1), the contrast was gradually lowered on-the-fly according to individual performance. We manipulated the contrast to increase the difficulty of the training and motivate participants to pay more attention to discriminating between pattern and oddball stimuli.

The types of oddball stimuli included images of simple forms; e.g., squares of different colors (one color per image), simple objects (such as a red or yellow ball, a green tennis ball, or a red apple), and more complex objects (e.g., the head of Hello Kitty, SpongeBob SquarePants, Mickey Mouse, or different kinds of cars). Objects were presented in color or in black-and-white and with high or low contrast. The selection criteria of oddball stimuli were based on the child’s acuity, preferences, and training goals. If a child had severe VA impairment or inattention, large and colorful oddball stimuli were used. If a child had better acuity and attention, small and low-contrast chromatic or achromatic oddball stimuli were used, as these were closer to the properties (size and contrast) of the checks in the checkerboard pattern. Random oddball stimuli were superimposed on the checkerboard patterns with a frequency of 15%, and participants were required to press an adaptive single switch immediately after detecting them. The oddball stimuli remained onscreen for 3 s, 2 s, 1.5 s, or 0.8 s. For children with severe visual impairment, short attention spans, and motor impairment, we increased the stimulus duration to allow a longer response time window. Different auditory feedback signals were used for a correct response (pressing the special switch for an oddball stimulus) and for an incorrect response (pressing the switch for a checkerboard pattern).

### Outcome Measures

Visual acuity, daily visual function, and VEP were assessed for each participant before and after the visual training. The VA and VEP assessments were scheduled on different days. The VA was always assessed before the VEP testing.

#### Visual Acuity

Visual acuity was assessed with a set of Teller acuity cards (TAC; Teller et al., 1986; Vistech Consultants, Inc., Dayton, OH, USA) for near (38 cm) or distant (84 cm) acuity, or with the Lea symbols Folding 15 Line distance chart for near (40 cm) or distant (1.5 m) acuity (Hered et al., 1997). For children under 3 years of age or children with difficulty identifying Lea symbols, the TAC was used. The testing distance was set at 84 cm for the TAC and 1.5 m for the Lea Symbols chart at first. If the child could not discriminate or recognize the stimuli, the test distance was changed to 38 cm for the TAC and 40 cm for the Lea Symbols chart to decrease the testing time and the burden on the participants. Results of the TAC test were converted from the unit of cycles/cm to the Snellen equivalent, a decimal scale, and finally the logMAR scale for comparison and statistical analysis. The luminance of both card sets was maintained at approximately 92 cd/m^2^, measured with a Minolta LS-110 luminance meter.

#### Functional Vision

Daily visual function was measured with the Functional Vision Questionnaire (FVQ), which is designed to evaluate visual function in children with cerebral palsy; it is also suitable for other preverbal children (Ferziger et al., 2011). The FVQ contains questions for assessment of visual responses in a lit room and in a darkened room, and for visual function related to communication, activities of daily life, and orientation and mobility (Ferziger et al., 2011). The original edition of the FVQ was translated with forward and backward translation into a traditional Chinese version with the permission of the original author. The full score of the Chinese-version FVQ is 140 points.

#### VEP Testing

Electroencephalograms (EEGs) were recorded with a high-density, 256-channel HydroCell Sensor Net system (Electrical Geodesics Inc., Eugene, OR, USA). The reference was located at the vertex, and electrode impedance was kept below 80 kΩ. EEG signals were acquired and band-pass filtered from 0.1 to 100 Hz at a sampling rate of 1,000 Hz. For EEG recording, the participant sat in a dimly lit room, 0.5 m in front of a CRT display. The visual stimulus of the black-and-white checkerboard pattern was phase reversed at 1 Hz for a total of 150 times per session. The same session was repeated three times. The checkerboard stimulus–with checks of 2° in size–subtended a visual angle of 8° vertically and 6° horizontally on either side of the fixation point. The mean luminance was 29 cd/m^2^, and the gamma value was 2.54. A contrast level of 95% was defined by the Michelson contrast formula. Participants were instructed to fixate on a cross marker with 1° of visual angle in the center of the screen. Monocular or binocular PVEPs were recorded depending on the eye being trained.

Electroencephalogram signals were analyzed off-line with Net station 4.3 and the EEGLAB toolbox. The EEG signals were first filtered using a 60 Hz Notch filter. Next, the channels contaminated by significant eye movement and eye blink which were prominent in young and behaviorally difficult to manage children and other notable bad channels were identified by visual inspection and removed. Then these bad channels were replaced with interpolated values from the surrounding electrodes. After that, all EEG signals were re-referenced to the average reference. Time windows of 50 ms before and 450 ms after were used to extract data epochs. The criterion of artifact rejection in epoched data was based on the extreme values (±150 μV). Good data epochs were baseline corrected (baseline from –50 to 0 ms). Finally, visual inspection was applied again to check and remove the remaining artifact activity. The EEG data were analyzed from the Oz electrode position to compare the differences pre- and post-training.

### Training Procedures

Visual training was conducted by an experienced occupational therapist. Over 1 month, participants received eight rounds of training: two rounds per week of at least eight sessions per round. We provided more training sessions for children with good attention and cooperation, and fewer sessions for the others. Each session consisted of 200 (for children with difficulty maintaining attention to the task) or 300 reversals, so each session would take between 1 and 5 min, depending on the reversal rates, whether a slower rate (2 Hz) or a higher rate (4 or 5 Hz), and the numbers of reversals. The time interval between any two sessions was adjusted according to each child’s condition and needs. Generally, each participant was allowed to rest for 1 to 2 min between sessions and for 5 to 10 min after finishing 4 to 5 sessions.

The training was conducted in a dim room. The participant sat on a height adjustable chair at a viewing distance of 30, 50, or 100 cm, depending on the child’s visual capacity. The visual training for every session simultaneously included a “fixation task” and a “detection task.” The checkerboard patterns were presented before the oddball stimuli, and the oddball stimuli were randomly superimposed on the checkerboard patterns with a frequency of 15%. In the fixation task, the children were encouraged to maintain fixation on the center of the screen, on which a checkerboard pattern of predetermined size, contrast, and reversal rate was continuously presented. In the detection task, which featured increasing attentional modulation of visual processing, participants were required to maintain the fixation task while simultaneously detecting the presence of the predetermined oddball stimuli. Participants were also instructed to press the special switch in response to an oddball stimulus as fast and as accurately as possible. In addition to auditory feedback triggered by pressing the switch, verbal cues or physical prompts were also given frequently at first to help participants to stay on task and then slowly removed to achieve the least invasive condition.

### Statistical Analysis

The results were mainly presented in the form of a case report. However, statistical analysis was also used to examine the effect of our VS program. The Wilcoxon signed-rank test was used to compare the scores of VA and the FVQ before and after visual training. The values of VA were first transformed into a decimal scale (for the TAC test) and then converted to a logMAR scale for analysis (for the TAC and Lea symbols tests). Statistical analyses were performed using SPSS 13.0 for Windows (SPSS Inc., Chicago, IL, USA).

## Results

### Participant Summary

Fourteen children with visual problems were referred to this study. Two of them could not follow simple commands, one had difficulty in maintaining attention, and the main caregivers of four of them were unable to participate in this study continuously for the full period. In the end, seven children were recruited for the study, and six of them (three females, three males; mean age = 3.9 ± 2.3 years, range = 1.2–7.8 years) finished the entire intervention program. One child dropped out after initial visual assessment; no definite reasons were provided by this child’s mother. **Table 1** summarizes participants’ demographic and medical information: age, gender, diagnosis, and refractive errors.

Three of the six children had retinal diseases, two were diagnosed with amblyopia, and one had cortical visual impairment (CVI). Case A3, case A5, and case A6 were referred by a special organization for people who have sensory, physical, or intellectual disabilities. Case A1 (male, 1.2 years old) was born premature at 25 weeks of gestation, with an extremely low birth weight (772 g). Stage 5 retinopathy of prematurity (ROP) in the right eye and stage 4B ROP in the left eye developed at 37 weeks. Intervention with Avastin injection, vitrectomy, and cataract surgery on both eyes were used to treat the progressive ROP problems. The retina of the left eye then reattached, although a ridge and a pulled-up retina were also observed. In addition, case A1 also had mild hearing impairment with the correction of hearing aids and global developmental delay (data from another early intervention centre). The visual impairment of case A2 (female, 2.3 years old) resulted from a subdural hemorrhage following a head injury at the age of 20 months. The VA of her left eye, with retinal detachment, was worse than that of her right eye, so the left eye was selected as the training eye. In case A3 (female, 3.2 years old), general delay with emotional behavior disorder, short attention span, suspected autistic disorder, and amblyopia were reported. For case A4 (male, 3.9 years old), binocular retinoschisis, nystagmus, hyperopia, and coordination disorder were reported. He also showed mild attention deficits. For case A5, who had binocular amblyopia and developmental delay, a daily 4-h dose of right eye patching was recommended by her ophthalmologist. Because of poor compliance, visual training of the left eye was substituted for patching during the study period. Case A6 (male, 7.8 years old) was born premature and diagnosed with spastic quadriplegia cerebral palsy (Level 4 of the Gross Motor Function Classification System) and CVI with damage to the periventricular white matter while participating in this study. Comprehensive developmental assessment was not performed in this study, including assessing their motor skills. The required hand movement was to press the special switch for the appearance of oddball stimuli. Only case 6, who had spastic CP, sometimes had difficulties in motor control and could not quickly respond to oddball stimuli; this child needed physical assistance to press the switch. All cases but case A2 (2.3 years old) and case A4 had other developmental problems. Case A4 also exhibited attention deficits and needed a lot of verbal promotion to help him to concentrate on a task. Therefore, these children, due to age or to additional developmental problems, had difficulty in performing the VPL task. That was why these children were included in this study.

Children with similar degrees of VA between the right and left eyes (cases A3, A4, and A6) received training under the binocular condition. Because the right eye of case 1 had little response to light, he also received training under the binocular condition. More information about the training eye is provided in the **Table 2**.

**Table 2:** Characteristics of checkerboard pattern and oddball stimuli for visual training (*n* = 6).

Case	Training eye(s)	Check size estimation (°)	Check size first vs. last training (°)	Check contrast first vs. last training (%)	Oddball size first vs. last training (°)
A1	OU	12.0	10→4	90→80	70→20
A2	OS	1.56	12→6	80→80	60→15
A3	OU	0.10	4→1	80→40	12→4
A4	OU	0.20	8→3	80→40	30→6
A5	OS	0.10	4→2	80→40	8→1
A6	OU	0.05	6→1	80→40	6→1

*Check size estimation means that the check size was estimated based on the child’s VA value (Iyer et al., 2013). “Check size first vs. last training (°)” means the check sizes used at the start and the last sessions of training. The unit for check size was degree of visual angle. The size and contrast were gradually decreased as the performance of the participants improved. Shown here are only the first and the last values. Contrast for checkerboard pattern is defined as Michelson contrast, for which the formula is (*I*_*max*_ – *I*_*min*_)/(*I*_*max*_ + *I*_*min*_).*

### Training Stimuli Summary

The mean number of pattern reversals was 3,096 reversals (*SD* = 765), and oddball stimuli were presented 451 times (*SD* = 85) in each session. **Table 2** shows the summarized individual stimuli parameters of training, including the initial estimated check sizes, actual check sizes (the first vs. the last size for training), check contrasts, and oddball sizes of six participants. The actual training check sizes were larger than the estimated sizes according to the relationships between VA and pattern sizes (Iyer et al., 2013). Because most of our participants had severe visual impairment or attention deficits, they had difficulty maintaining attention on small check sizes, which were converted from the threshold of the smallest stimulus size to discriminate or recognize (definition of VA). Therefore, a larger check size was needed for our participants to maintain fixation on the screen for the purpose of passive VS. Rough criteria to determine the size of oddball stimuli were used in current study. For example, when the case had severe visual impairment and short fixation time, such as case A1 and case A2, large oddball stimuli (e.g., 60 or 70° of visual angle) were used at the beginning to make sure that the children could easily see the oddball stimuli. As they progressed with better visual responses (maintaining fixation with little assistance), the size and contrast for the checks and the oddball stimuli were gradually decreased.

### Visual Acuity and Functional Vision

**FIGURE 1:**
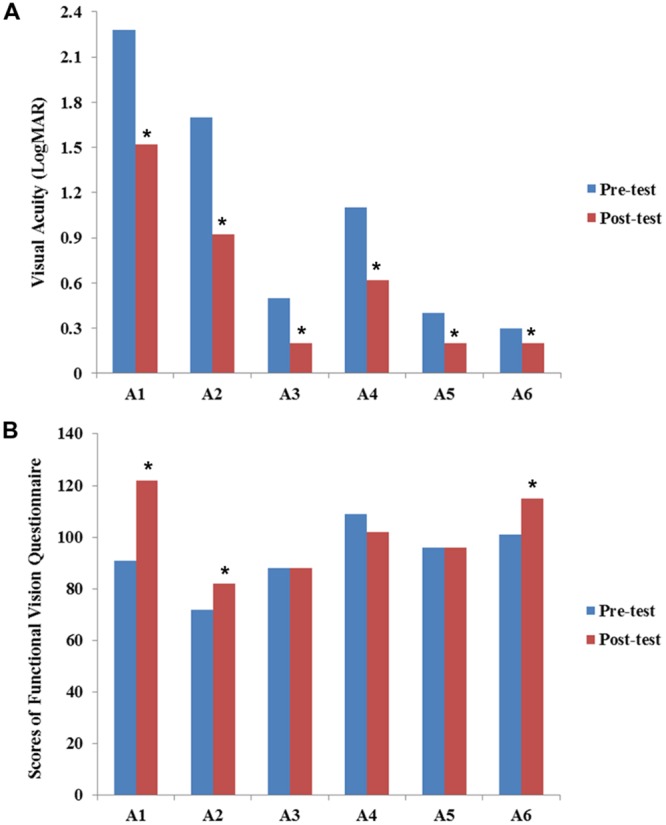
**Results of visual acuity **(A)** and functional vision **(B)** before and after the VS program.** Values of visual acuity and functional vision assessment are individually presented for six participants (A1–A6). Data are presented for pre-training (blue bars) and post-training (red bars). The scores of visual acuity are transferred and expressed in LogMAR scale. All participants showed improvement in VA after visual training. Statistical analysis also revealed significant differences between pre-VS and post-VS [*Z* = –2.20, asymptotic significance (2-tailed) = 0.028]. The Chinese version of the functional vision questionnaire was used to assess functional vision (full score = 140 points). Although some participants showed improvement in functional vision, no statistically significant difference was found [*Z* = –1.46, asymptotic significance (2-tailed) = 0.144]. Asterisk (^∗^) indicates improvement in the scores of VA and functional vision after VS program.

The results of VA for six participants assessed at baseline and post-training expressed in LogMAR scale are presented in **Figure 1A**. All participants showed improvement in VA after our visual training program.

#### Case 1

The participant’s right eye had light perception, and the left eye was his better eye. Unstable binocular performance (<50% accuracy) on the Teller acuity low vision (LV) card (0.23 cycles/cm) at a viewing distance of 15 cm was observed before training (recorded as LV not seen), but he could detect 0.64 cycles/cm at a test distance of 38 cm (approximated to 0.03 in decimal notation) after training (Teller et al., 1986). Because A1 had VA of “hand movement,” a decimal VA of 0.0052 was noted for further analysis (Lange et al., 2009).

#### Case 2

In case A2, a subdural hemorrhage caused severe retinal hemorrhage and diminished the left eye’s VA. She had an acuity of 0.86 cycle/cm at a test distance of 20 cm (equal to 0.02 in decimal notation) and could not detect the low vision strip card at 84 cm (distance for distance acuity suggested by the Teller acuity test). However, she was able to discriminate strips with 2.4 cycles/cm at a distance of 38 cm (equal to 0.12 in decimal notation), and a low vision card at 84 cm after training.

#### Case 3

Because she could binocularly discriminate the finest Teller card with 38 cycles/cm strips at a test distance of 84 cm, the Lea Symbols VA test was used to assess her acuity. In terms of VA before and after training, she was able to recognize the Lea symbols of the 6.0 M-unit (M) size (pre-training, equal to 0.32 in decimal notation) and the 2.4 M size (post-training, equal to 0.63 in decimal notation) at a viewing distance of 1.5 m.

#### Case 4

This participant had the same near acuity before and after training (TAC with 13.0 cycles/cm strips at a test distance of 38 cm). However, his distance acuity improved from 1.6 cycles/cm (equal to 0.08 in decimal notation) to 4.8 cycles/cm strips (equal to 0.24 in decimal notation) at a viewing distance of 84 cm. Although he could identify fine strips after visual training, he still could not recognize the 30 M size of Lea symbols at a 1.5 m test distance (equal to 0.05 in decimal notation).

#### Case 5

Her binocular VA, right eye VA, and left eye VA enabled this participant to recognize the 3.0 M size, 3.0 M size (about 0.50 in decimal notation), and 3.8 M size (about 0.40 VA in decimal notation) of Lea symbols at a test distance of 1.5 m, respectively. Her program was right eye patching to enhance her left eye’s acuity. After training, not only the left eye VA but also the binocular and right eye VA showed improvement, and she was able to recognize symbols with sizes of 2.4 M (left eye, about 0.63 in decimal notation), 1.9 M (both eyes), and 1.9 M (right eye, about 0.80 in decimal notation).

#### Case 6

His distance acuities before and after visual training allowed this participant to recognize the 3.0 M (equal to 0.50 in decimal notation) and 2.4 M size (equal to 0.63 in decimal notation) Lea symbols at a distance of 1.5 m. Although his acuity was only one line better than at the pre-test, he could recognize the symbols more quickly.

The results of FVQ for the six participants assessed at baseline and post-training are presented in **Figure 1B**. Cases A1, A2, and A6 showed improvement in both VA and functional vision. For case A1, the main improvement was in the play and leisure areas. For example, his frequency of looking at a toy while he was reaching for it or when a toy was placed in his hand increased in frequency. Case A2 showed improvement in response to an object in a lit room or an illuminated object in a darkened room. The mother of case A6 reported that the participant had more visual response in communication with others, such as focusing on another’s face or responding to facial expressions. Case A6 also demonstrated improvement in responding to an object in a lit room. Individual results of VA and functional vision are also presented in the Supplementary Table 1.

In addition, the results of VA (pre-mean = 1.05 logMAR, *SD* = 0.80; post-mean = 0.61 logMAR, *SD* = 0.53) and the FVQ (pre-mean = 92.8, *SD* = 12.6; post-mean = 100.8, *SD* = 15.4) of the 6 participants were also combined for statistical analysis. The Wilcoxon Sign-Ranked Test results revealed significant differences between pre-VS and post-VS [*Z* = –2.20, asymptotic significance (2-tailed) = 0.028]. These differences indicated that VA improved significantly after the VS program. However, no significant improvement was found in the functional vision assessment [*Z* = –1.46, asymptotic significance (2-tailed) = 0.144].

### Pattern Reversal VEP

**FIGURE 2:**
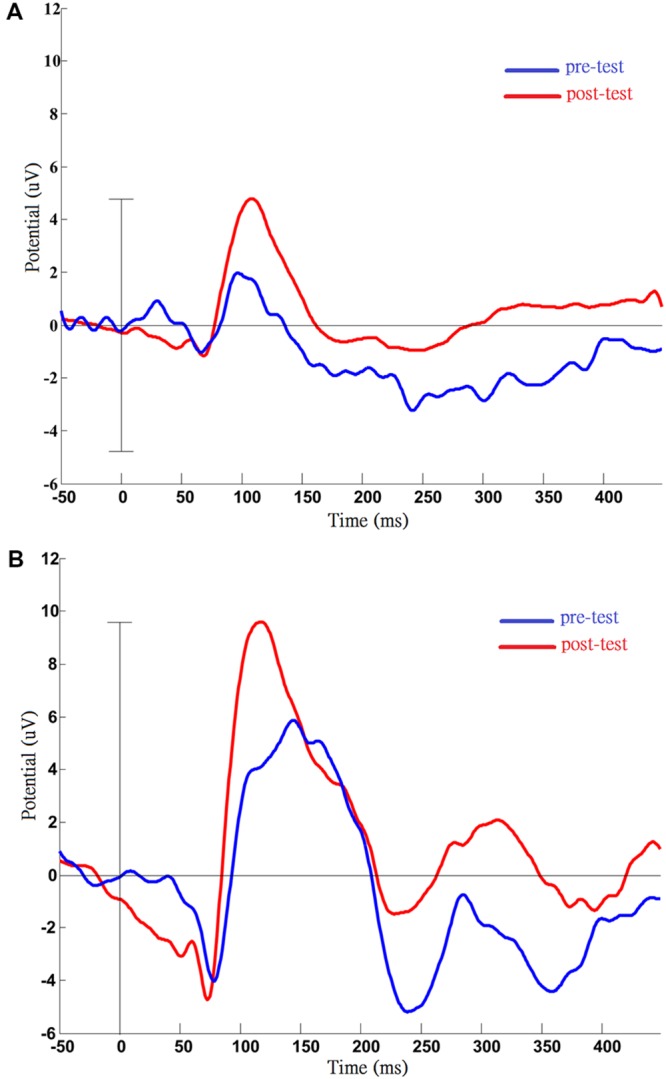
**Results of pattern visual evoked potential (VEP) testing before and after the VS program in case A5 **(A)** and case A6 **(B)**.** Pre- and post-measurements of VEP were completed by case A5 and case A6. After training, the VEP amplitude was increased compared with pre-test, and this phenomenon was persistent in two cases. **(A)** Data from case A5: increased P100 amplitude (pre: 1.97 uV; post: 4.78 uV); **(B)** Data from case A6: post-training, shortened latency (pre: 144 ms; post: 118 ms), and increased amplitude of P100 (pre: 5.85 uV; post: 9.59 uV).

For the pattern reversal VEP (PRVEP), four of the participants finished the pre-test (cases A1 and A3 strongly refused the EEG cap), and cases A4, A5, and A6 finished the post-test (case A2 resisted patching for the VEP test). In addition to retinoschisis, case A4 also had abnormal nystagmus, which caused the pattern reversal response to be absent. In the end, only data from cases A5 and A6 were analyzed to present the change before and after the visual training (**Figure 2A**: A5, **Figure 2B**: A6). For case A5, there were slight changes in the P100 latency (pre-test: 97 ms; post-test: 108 ms), but apparent changes in amplitudes (pre-test: 1.97 uV; post-test: 4.78 uV) were noted after training. For case A6, the P100 latency was shorter (pre-test: 144 ms; post-test 118 ms), P100 amplitude was increased (pre-test: 5.85 uV; post-test: 9.59 uV), after visual training.

## Discussion

In this study, we developed a new computer-based VS program to improve the VA of young children with visual impairment or those with impaired VA and other neurological disorders. Primitive pattern reversal, with variation in check size, contrast, and reversal rate, was used as the core VS, and different types of oddball stimuli, determined by the children’s visual capacities and preferences, were additionally integrated into this program to facilitate visual fixation and visual attention so as to increase the effect of visual training. The observed training outcomes in VA were promising even though the sample size was small, heterogeneous participants were included, and only limited numbers of training sessions were conducted.

Traditionally, a passive VS program is mainly applied to VI infants or children with CVI (Leguire et al., 1992; Malkowicz et al., 2006; Alimovic and Mejaski-Bosnjak, 2011) because they usually have difficulty in actively reacting to surrounding visual stimuli or participating in complex visual training activities. However, the results indicated that our modified VS program could benefit children across a wider range in terms of age, severity of visual impairment, and type of diagnosis. For example, case A6 (7.8 years old) was diagnosed with spastic cerebral palsy and CVI and had motor control problems and inattention. He therefore needed some physical assistance in operating the adaptive switch and more verbal prompts to remind him to pay attention to the task. For this older child, simple passive checkerboard stimulation and attention enhancement helped to improve his VA. The improvement in case A6 was also apparent in the changes of the VEP: P100 latency was shortened and P100 amplitude was increased. Therefore, for children who have difficulty in performing visual training tasks with higher task demands, such as VPL, this modified computer-based VS program could be an alternative to strengthen their VA.

The core passive stimulus used in this study was a high-contrast checkerboard pattern reversal. The findings of this study support the usefulness of such a primitive stimulus in visual training, which can be used in children of different ages and cognitive stages. High-contrast patterns and shapes are often used in developmental research on infant vision, since infants prefer black and white geometric shapes to bright colors (Downing and Bailey, 1990). High-contrast patterns that are primitive, simple, and reliable are also extensively used in visual science (De Valois et al., 1979), visual function assessment (e.g., pattern VEP), and visual training (Leguire et al., 1992; Malkowicz et al., 2006; Alimovic et al., 2013). In addition, in the visual cortex, the neurons are tuned to simple visual characteristics, such as spatial frequency, edges, contrast, and local motion (De Valois et al., 1979). Therefore, manipulation of the check size (spatial frequency), contrast, and reversal rate in a checkerboard pattern is useful in the VS program.

In not only our study but in previous VS studies (Werth and Moehrenschlager, 1999; Malkowicz et al., 2006; Alimovic and Mejaski-Bosnjak, 2011), and even in another visual training program for children with visual impairment (Sonksen et al., 1991), the content of visual training was adapted to the visual function of the VI infants or children. Although our study did not have the definite hierarchic stages of the study of Malkowicz et al. (2006), we graded our participants’ abilities in terms of check size, check contrast, and oddball size.

One of the significant modifications in our VS program was the addition of manipulation of top-down attentional demands into the visual training task (Grill-Spector and Malach, 2004). The inclusion of oddball stimuli, active response through the single adaptive switch, and interesting auditory feedback helped to increase active task participation, increase overall attention span for training, and enhance focused attention to facilitate learning effects. In addition, several previous studies have indicated differences in P300 responses between passive and active tasks (LaGrow et al., 1998; Vervloed et al., 2006) and the positive effect of feedback on performance and brain activation (Willshaw et al., 1980). Although passive oddball stimuli can also elicit P300 responses, their effects are weaker than those of an active oddball task (Vervloed et al., 2006). Thus, attention manipulation plays a key role in this VS program.

Another important factor in our design that led to improvements in VA after only eight rounds of visual training within 1.5 months may have been the high doses of repeated VS. The mean number of pattern reversals was 3,096 reversals (*SD* = 765), and oddball stimuli were presented 451 times (*SD* = 85) in each round. These numbers are far larger than those used in previous VS studies (Malkowicz et al., 2006; Alimovic et al., 2013). The results also provide a new alternative approach for visual rehabilitation for children with impaired VA. Further studies will be required to investigate the impact of both the frequency and doses of VS delivery on the development of visual function and functional vision.

Although no significant improvement in functional vision was observed in this study, positive changes were observed in some participants (A1, A2, and A6). The original purpose of the FVQ is to evaluate visual function in children with cerebral palsy and other preverbal children with visual impairment (Ferziger et al., 2011), so this test would be not appropriate for assessing children beyond the preverbal age and those with only mild developmental problems (such as cases A3, A4, and A5). Further studies adopting another age-appropriate FVQ, one designed for older children with visual problems, will be necessary to improve the sensitivity in detecting the functional changes after visual training.

The current study had several limitations. First, our sample size was small, and only a pre-test–post-test study design was adopted, which provided a lower level of evidence from which to formulate conclusions. Therefore, further studies using larger sample sizes and a randomized controlled trial to confirm the effects of this VS program as compared with those of other VS training paradigms are recommended. Second, in this pilot study, participants spanning a wide age range were included. Different age groups may have different responses to the VS training, so it is suggested that further studies include more participants and divide them into two or three separate age groups to avoid this issue. Third, the pattern reversal rates were 2 Hz for younger children and those with VA below 0.1, and 4 or 5 Hz for other children. However, it would be interesting to investigate whether the reversal rate can influence the perception of pattern reversal and the effects of training. Fourth, children with various visual impairments were included into this study. Although the purpose of this pilot study was only to investigate whether a VS program combining sensory stimulation and attention modulation could be effective, in spite of the lesions along with the anterior and/or poster pathways, further studies to identify whether there is a difference in visual responses, visual progressing, and even the mechanism behind visual change between the participants in brain or ocular alternations is required. Fifth, the most important feature of this VS program is that it combines constant checkerboard stimuli (bottom-up feedforward) and oddball stimuli (top-down modulation). However, from the current study and available data, it cannot be determined whether our VS program would be better than only providing VS, as in the Leguire et al. (1992) study. Furthermore, another important issue is how to adjust the ratio of checkerboard and oddball stimuli to make the visual training more efficient in further studies.

## Conclusion

This pilot study indicates that our VS program of integrating constant checkerboard stimulation (bottom-up feedforward) and random oddball stimulation (top-down modulation) may be helpful to improve the VA for VI children with additional developmental problems or those unable to cooperate with VPL training. For some of the children, the changes were also shown by the functional vision and VEP assessments after only eight rounds of VS training in 2 months. The sizes and contrast of the checkerboard and oddball stimuli were adjusted according to these children’s visual performance. Therefore, the content of this program was not constant, varying with each child’s condition. In addition, high doses of stimulation were emphasized in this study, which averaged over 3,000 checkerboard reversals and 400 occurrences of oddball stimulation in each round. However, the sample was small, various types of visual impairment caused by ocular and/or brain alternations and age groups were included, and no control group was used. Also, it is unknown whether our VS program is better than previous VS programs or better than only the checkerboard used in training. Therefore, several limitations should be resolved in further investigations before advanced clinical application.

## Author Contributions

L-TT designed the study, collected the data, analyzed the data, and wrote the manuscript. J-LH analyzed the VEP and reviewed and edited the manuscript. C-TW contributed to collecting the data and to reviewing and editing the manuscript. Y-CS contributed to the study design, facilitated data collection, assisted in the interpretation of the data, and reviewed and edited the manuscript. C-CC contributed to collecting data and carrying out VS training program. Y-CS is the guarantor of this work and, as such, had full access to all the data in the study and takes responsibility for the integrity of the data and the accuracy of the data analysis.

## Conflict of Interest Statement

The authors declare that the research was conducted in the absence of any commercial or financial relationships that could be construed as a potential conflict of interest.
