# Supplementary material for: A New Visual Stimulation Program for Improving Visual Acuity in Children with Visual Impairment: A Pilot Study
Source: Front Hum Neurosci. 2016 Apr 18;10:157. doi: 10.3389/fnhum.2016.00157 (PMC4834310; doi:10.3389/fnhum.2016.00157)
Supplement: Supplementary file 1 [file Table_1.DOCX]

***Supplementary Material***

**A new visual stimulation program for improving visual acuity in children with visual impairment: a pilot study**

Li-Ting Tsai, Jung-Lung Hsu, Chien-Te Wu, Chia-Ching Chen, Yu-Chin Su^*^

*** Correspondence:** Yu-Chin Su: [hermessu828@gmail.com](mailto:hermessu828@gmail.com)

# Supplementary Table

**Supplementary Table 1. Individual performance of visual acuity and functional vision in six participants before and after the VS program.**

| **Subject** | **Sex** | **Age, y** | **Diagnosis** | **Training eye(s)** | **Visual acuity** | **Functional vision** |
| --- | --- | --- | --- | --- | --- | --- |
| **A1** | M | 1.2 | ROP (stage 5 in OD and 4 in OS) & Aphakia (OU) & hearing impairment | OU | **Pre:** Light perception in right eye. Left eye is the better eye. Unstable binocular VA on the Teller acuity low vision card (0.23 cycles/cm) at 15 cm.  **Post:** 0.64 cycles/cm of the Teller acuity card of binocular VA at 38 cm. | **Pre:** FVQ score was 91.  **Post:** FVQ score was 122.  Main improvement was in the play and leisure areas; e.g., looking at a toy while he was reaching for it. |
| **A2** | F | 2.3 | Subdural hemorrhage with retinal hemorrhage in OD & retinal detachment in OS | OS | **Pre:** VA in OS was 0.86 cycles/cm of the Teller acuity card at viewing distance of 20 cm.  **Post:** VA in OS was 2.4 cycles/cm of the Teller acuity card at 38 cm, and low vision card at 84 cm. | **Pre:** FVQ score was 72.  **Post:** FVQ score was 82.  Improvement was shown in response to an object in a lit room or an illuminated object in a darkened room. |
| **A3** | F | 3.2 | Suspected autistic disorder with amblyopia (OU) | OU | **Pre:** Recognizing 6.0 M of symbols of the Lea Symbols VA test at a distance of 1.5 m.  **Post:** Recognizing 2.4 M of symbols of the Lea Symbols VA test at a distance of 1.5 m. | **Pre:** FVQ score was 88.  **Post:** FVQ score was 88. |
| **A4** | M | 3.9 | Retinoschisis (OU) | OU | **Pre:** Near VA was 13.0 cycles/cm of Teller acuity card at 38 cm. Distance VA was 1.6 cycles/cm at 84 cm.  **Post:** Near VA was the same. Distance VA was 4.8 cycles/cm of the Teller acuity card at 84 cm. | **Pre:** FVQ score was 109.  **Post:** FVQ score was 102. |
| **A5** | F | 5.3 | Premature birth with  amblyopia (OU) | OS | **Pre:** OS, OD, and OU VA were 3.8 M, 3.0 M, and 3.0 M of the Lea Symbols VA test at a distance of 1.5 m.  **Post:** OS VA was 2.4M at 1.5 m. OD and OU VA were also improved to recognize 1.9 M and 1.9 M symbols. | **Pre:** FVQ score was 96.  **Post:** FVQ score was 96. |
| **A6** | M | 7.8 | Cerebral palsy with cerebral visual impairment | OU | **Pre:** Recognizing 2.4 M of symbols of the Lea Symbols VA test at a testing distance of 1.5 m.  **Post:** Recognizing 1.5 M of symbols of the Lea Symbols VA test at a distance of 1.5 m. | **Pre:** FVQ score was 101.  **Post:** FVQ score was 115.  Showed more visual response in communication with others, and to an object in a lit room environment. |

*VA: Visual acuity, M: M-unit, Performance of functional vision was assessed by the Chinese-version Functional Vision Questionnaire (FVQ). The full score of the Chinese-version FVQ is 140 points.
